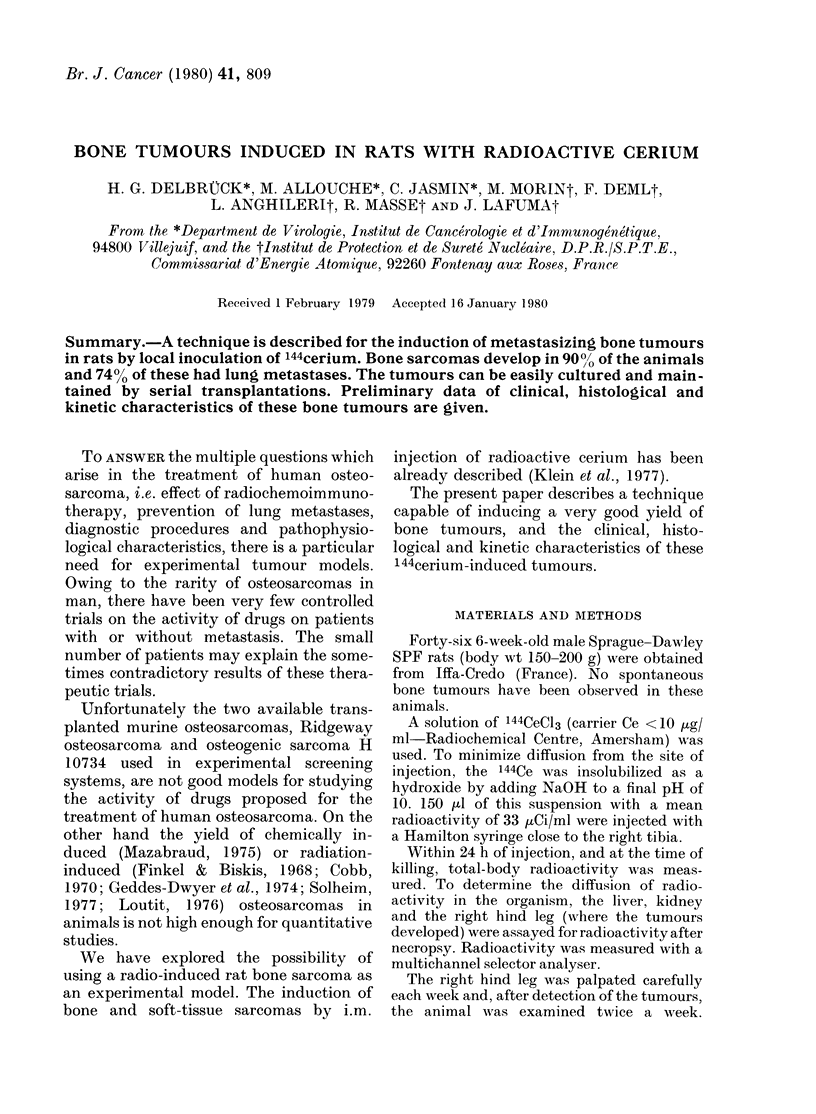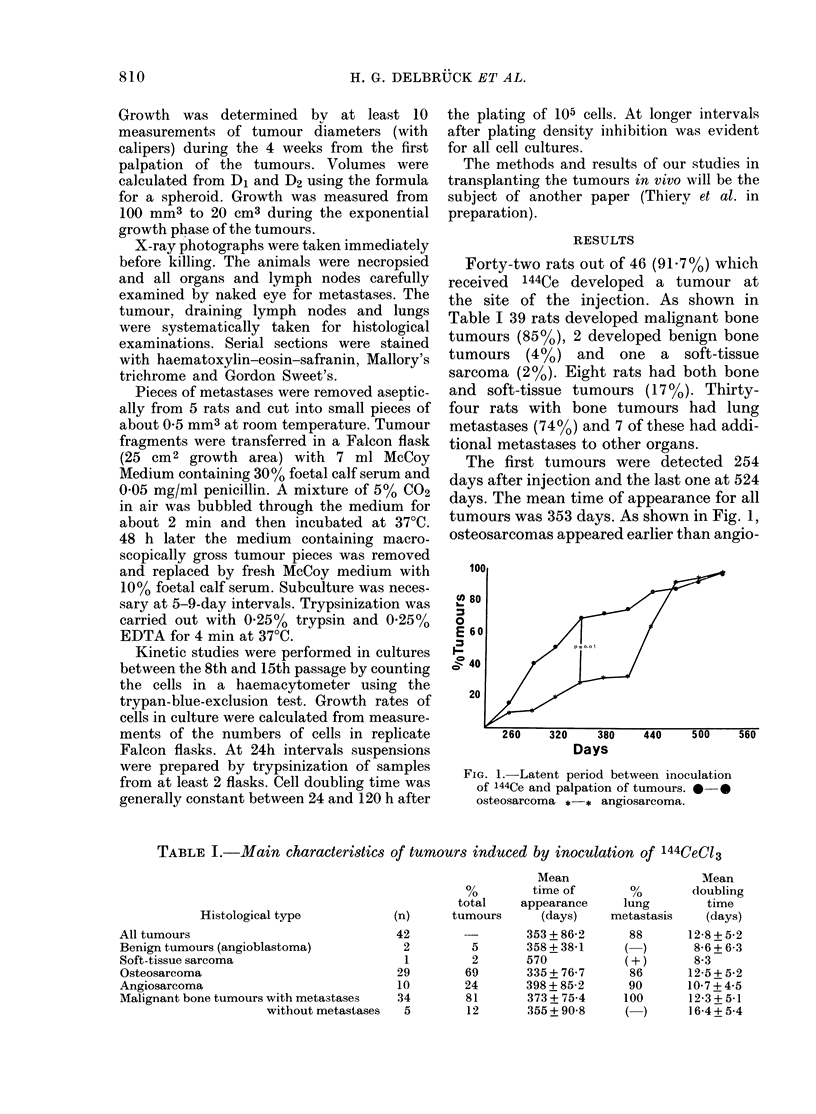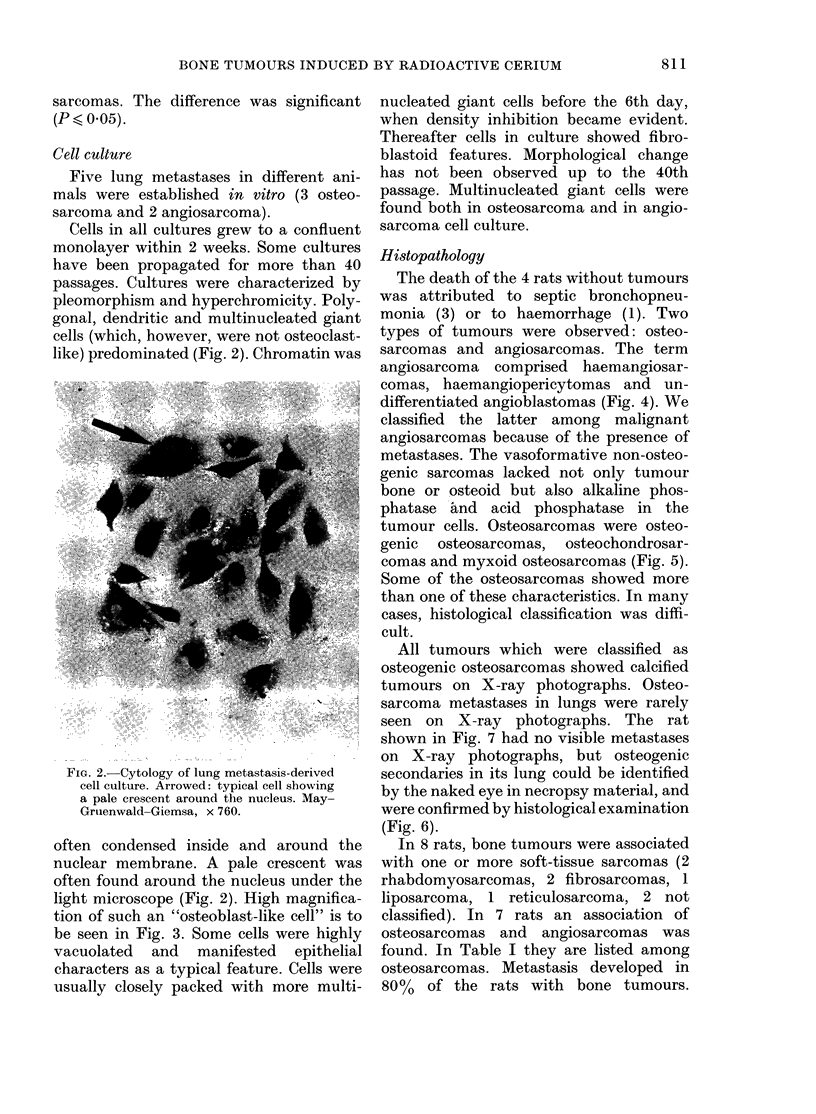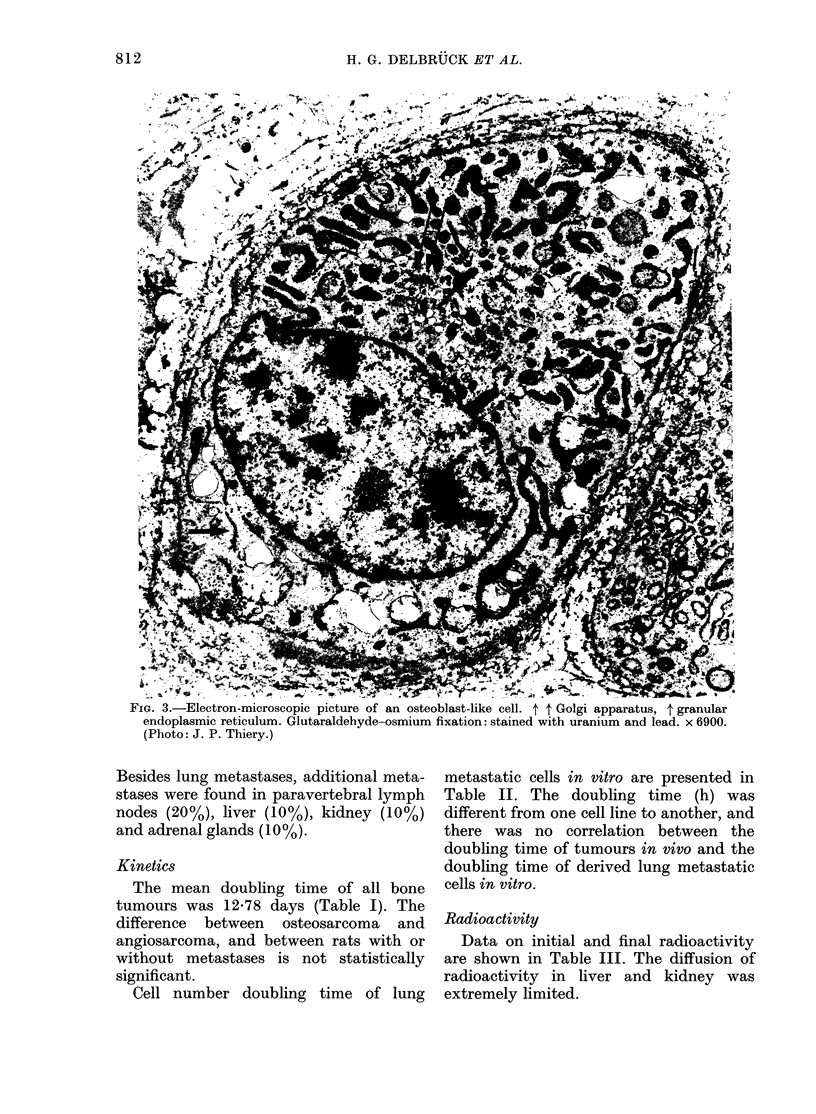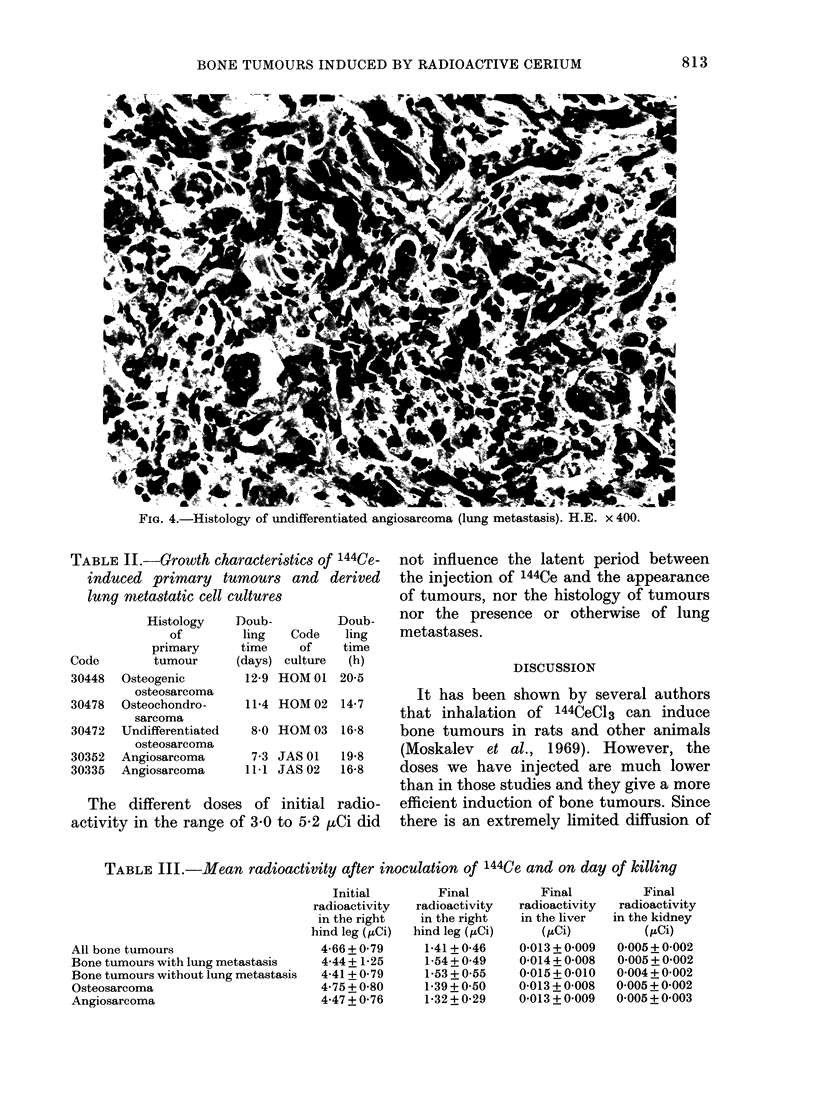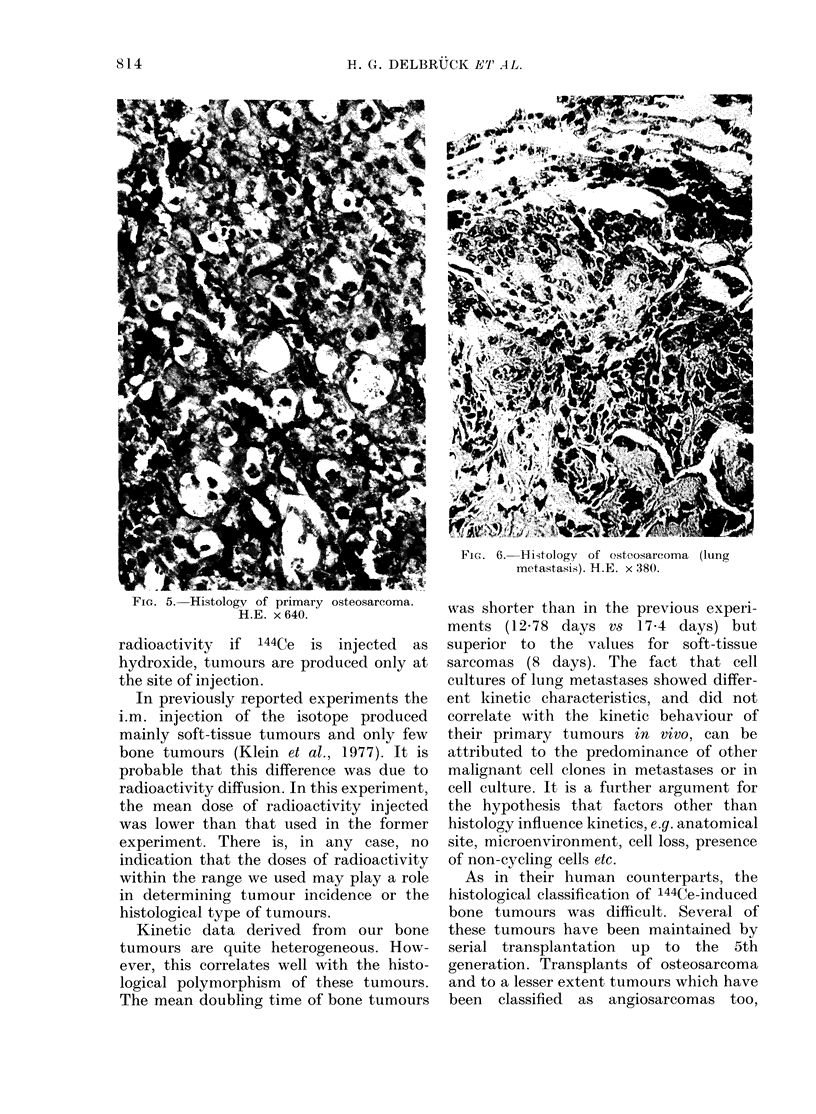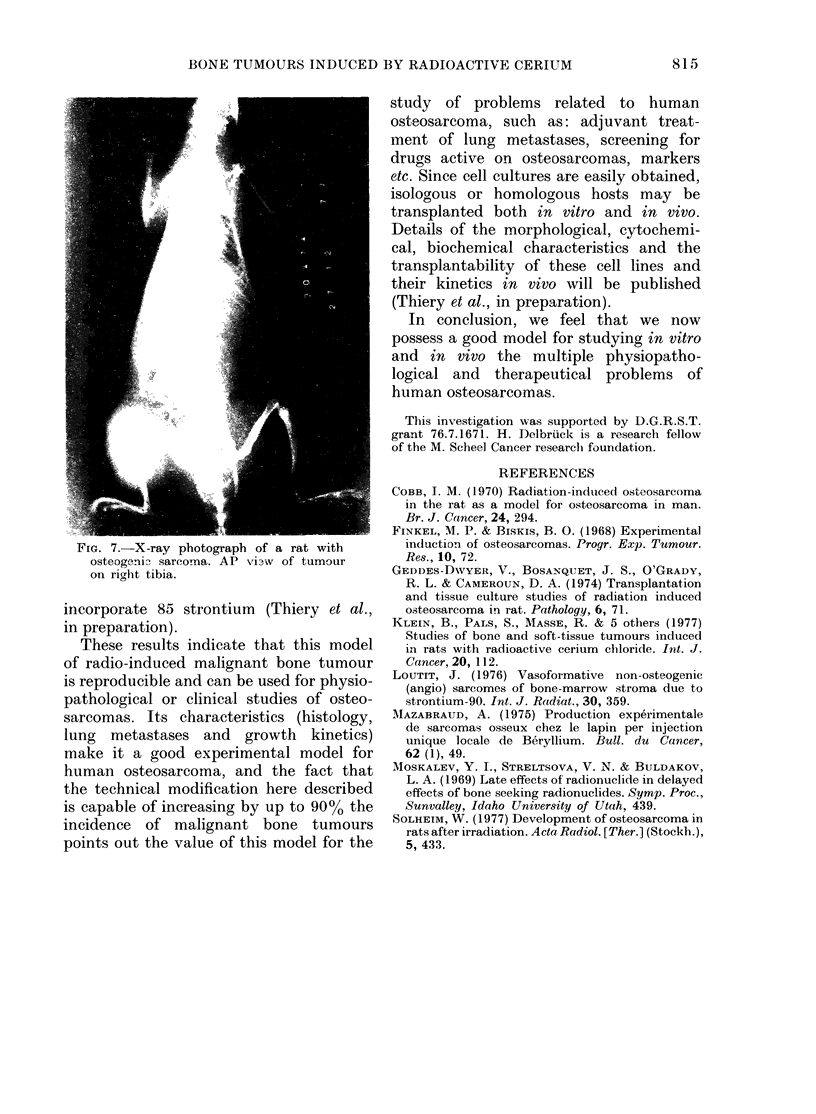# Bone tumours induced in rats with radioactive cerium.

**DOI:** 10.1038/bjc.1980.145

**Published:** 1980-05

**Authors:** H. G. Delbrück, M. Allouche, C. Jasmin, M. Morin, F. Deml, L. Anghileri, R. Masse, J. Lafuma

## Abstract

**Images:**


*Br. J. Cancer* (1980) **41**, 809

### BONE TUMOURS INDUCED IN RATS WITH RADIOACTIVE CERIUM

H. G. DELBRÜCK*, M. ALLOUCHE*, C. JASMIN*, M. MORIN†, F. DEML†, L. ANGHILERI†, R. MASSE† AND J. LAFUMA†

*From the *Department de Virologie, Institut de Cancérologie et d'Immunogénétique,* 94800 *Villejuif, and the †Institut de Protection et de Sureté Nucléaire, D.P.R./S.P.T.E., Commissariat d'Energie Atomique,* 92260 *Fontenay aux Roses, France*

Received 1 February 1979 Accepted 16 January 1980

**Summary.—A technique is described for the induction of metastasizing bone tumours in rats by local inoculation of $^{144}$cerium. Bone sarcomas develop in 90% of the animals and 74% of these had lung metastases. The tumours can be easily cultured and maintained by serial transplantations. Preliminary data of clinical, histological and kinetic characteristics of these bone tumours are given.**

TO ANSWER the multiple questions which arise in the treatment of human osteosarcoma, *i.e.* effect of radiochemoimmunotherapy, prevention of lung metastases, diagnostic procedures and pathophysiological characteristics, there is a particular need for experimental tumour models. Owing to the rarity of osteosarcomas in man, there have been very few controlled trials on the activity of drugs on patients with or without metastasis. The small number of patients may explain the sometimes contradictory results of these therapeutic trials.

Unfortunately the two available transplanted murine osteosarcomas, Ridgeway osteosarcoma and osteogenic sarcoma H 10734 used in experimental screening systems, are not good models for studying the activity of drugs proposed for the treatment of human osteosarcoma. On the other hand the yield of chemically induced (Mazabraud, 1975) or radiation-induced (Finkel & Biskis, 1968; Cobb, 1970; Geddes-Dwyer *et al.*, 1974; Solheim, 1977; Loutit, 1976) osteosarcomas in animals is not high enough for quantitative studies.

We have explored the possibility of using a radio-induced rat bone sarcoma as an experimental model. The induction of bone and soft-tissue sarcomas by i.m. injection of radioactive cerium has been already described (Klein *et al.*, 1977).

The present paper describes a technique capable of inducing a very good yield of bone tumours, and the clinical, histological and kinetic characteristics of these $^{144}$cerium-induced tumours.

### MATERIALS AND METHODS

Forty-six 6-week-old male Sprague–Dawley SPF rats (body wt 150–200 g) were obtained from Iffa-Credo (France). No spontaneous bone tumours have been observed in these animals.

A solution of $^{144}CeCl_3$ (carrier Ce $<10$ $\mu$g/ml—Radiochemical Centre, Amersham) was used. To minimize diffusion from the site of injection, the $^{144}$Ce was insolubilized as a hydroxide by adding NaOH to a final pH of 10. 150 $\mu$l of this suspension with a mean radioactivity of 33 $\mu$Ci/ml were injected with a Hamilton syringe close to the right tibia.

Within 24 h of injection, and at the time of killing, total-body radioactivity was measured. To determine the diffusion of radioactivity in the organism, the liver, kidney and the right hind leg (where the tumours developed) were assayed for radioactivity after necropsy. Radioactivity was measured with a multichannel selector analyser.

The right hind leg was palpated carefully each week and, after detection of the tumours, the animal was examined twice a week.

Growth was determined by at least 10 measurements of tumour diameters (with calipers) during the 4 weeks from the first palpation of the tumours. Volumes were calculated from $D_1$ and $D_2$ using the formula for a spheroid. Growth was measured from 100 $mm^3$ to 20 $cm^3$ during the exponential growth phase of the tumours.

X-ray photographs were taken immediately before killing. The animals were necropsied and all organs and lymph nodes carefully examined by naked eye for metastases. The tumour, draining lymph nodes and lungs were systematically taken for histological examinations. Serial sections were stained with haematoxylin–eosin–safranin, Mallory's trichrome and Gordon Sweet's.

Pieces of metastases were removed aseptically from 5 rats and cut into small pieces of about 0·5 $mm^3$ at room temperature. Tumour fragments were transferred in a Falcon flask (25 $cm^2$ growth area) with 7 ml McCoy Medium containing 30% foetal calf serum and 0·05 mg/ml penicillin. A mixture of 5% $CO_2$ in air was bubbled through the medium for about 2 min and then incubated at 37°C. 48 h later the medium containing macroscopically gross tumour pieces was removed and replaced by fresh McCoy medium with 10% foetal calf serum. Subculture was necessary at 5–9-day intervals. Trypsinization was carried out with 0·25% trypsin and 0·25% EDTA for 4 min at 37°C.

Kinetic studies were performed in cultures between the 8th and 15th passage by counting the cells in a haemacytometer using the trypan-blue-exclusion test. Growth rates of cells in culture were calculated from measurements of the numbers of cells in replicate Falcon flasks. At 24h intervals suspensions were prepared by trypsinization of samples from at least 2 flasks. Cell doubling time was generally constant between 24 and 120 h after the plating of $10^5$ cells. At longer intervals after plating density inhibition was evident for all cell cultures.

The methods and results of our studies in transplanting the tumours *in vivo* will be the subject of another paper (Thiery *et al.* in preparation).

### RESULTS

Forty-two rats out of 46 (91·7%) which received $^{144}Ce$ developed a tumour at the site of the injection. As shown in Table I 39 rats developed malignant bone tumours (85%), 2 developed benign bone tumours (4%) and one a soft-tissue sarcoma (2%). Eight rats had both bone and soft-tissue tumours (17%). Thirty-four rats with bone tumours had lung metastases (74%) and 7 of these had additional metastases to other organs.

The first tumours were detected 254 days after injection and the last one at 524 days. The mean time of appearance for all tumours was 353 days. As shown in Fig. 1, osteosarcomas appeared earlier than angio-

FIG. 1.—Latent period between inoculation of $^{144}Ce$ and palpation of tumours. ●—● osteosarcoma *—* angiosarcoma.

TABLE I.—*Main characteristics of tumours induced by inoculation of* $^{144}CeCl_3$

| Histological type | (n) | % total tumours | Mean time of appearance (days) | % lung metastasis | Mean doubling time (days) |
|---|---|---|---|---|---|
| All tumours | 42 | — | 353 ± 86·2 | 88 | 12·8 ± 5·2 |
| Benign tumours (angioblastoma) | 2 | 5 | 358 ± 38·1 | (—) | 8·6 ± 6·3 |
| Soft-tissue sarcoma | 1 | 2 | 570 | (+) | 8·3 |
| Osteosarcoma | 29 | 69 | 335 ± 76·7 | 86 | 12·5 ± 5·2 |
| Angiosarcoma | 10 | 24 | 398 ± 85·2 | 90 | 10·7 ± 4·5 |
| Malignant bone tumours with metastases | 34 | 81 | 373 ± 75·4 | 100 | 12·3 ± 5·1 |
| without metastases | 5 | 12 | 355 ± 90·8 | (—) | 16·4 ± 5·4 |

sarcomas. The difference was significant ($P \leqslant 0{\cdot}05$).

*Cell culture*

Five lung metastases in different animals were established *in vitro* (3 osteosarcoma and 2 angiosarcoma).

Cells in all cultures grew to a confluent monolayer within 2 weeks. Some cultures have been propagated for more than 40 passages. Cultures were characterized by pleomorphism and hyperchromicity. Polygonal, dendritic and multinucleated giant cells (which, however, were not osteoclast-like) predominated (Fig. 2). Chromatin was

FIG. 2.—Cytology of lung metastasis-derived cell culture. Arrowed: typical cell showing a pale crescent around the nucleus. May–Gruenwald–Giemsa, × 760.

often condensed inside and around the nuclear membrane. A pale crescent was often found around the nucleus under the light microscope (Fig. 2). High magnification of such an "osteoblast-like cell" is to be seen in Fig. 3. Some cells were highly vacuolated and manifested epithelial characters as a typical feature. Cells were usually closely packed with more multinucleated giant cells before the 6th day, when density inhibition became evident. Thereafter cells in culture showed fibroblastoid features. Morphological change has not been observed up to the 40th passage. Multinucleated giant cells were found both in osteosarcoma and in angiosarcoma cell culture.

*Histopathology*

The death of the 4 rats without tumours was attributed to septic bronchopneumonia (3) or to haemorrhage (1). Two types of tumours were observed: osteosarcomas and angiosarcomas. The term angiosarcoma comprised haemangiosarcomas, haemangiopericytomas and undifferentiated angioblastomas (Fig. 4). We classified the latter among malignant angiosarcomas because of the presence of metastases. The vasoformative non-osteogenic sarcomas lacked not only tumour bone or osteoid but also alkaline phosphatase and acid phosphatase in the tumour cells. Osteosarcomas were osteogenic osteosarcomas, osteochondrosarcomas and myxoid osteosarcomas (Fig. 5). Some of the osteosarcomas showed more than one of these characteristics. In many cases, histological classification was difficult.

All tumours which were classified as osteogenic osteosarcomas showed calcified tumours on X-ray photographs. Osteosarcoma metastases in lungs were rarely seen on X-ray photographs. The rat shown in Fig. 7 had no visible metastases on X-ray photographs, but osteogenic secondaries in its lung could be identified by the naked eye in necropsy material, and were confirmed by histological examination (Fig. 6).

In 8 rats, bone tumours were associated with one or more soft-tissue sarcomas (2 rhabdomyosarcomas, 2 fibrosarcomas, 1 liposarcoma, 1 reticulosarcoma, 2 not classified). In 7 rats an association of osteosarcomas and angiosarcomas was found. In Table I they are listed among osteosarcomas. Metastasis developed in 80% of the rats with bone tumours.

FIG. 3.—Electron-microscopic picture of an osteoblast-like cell. ↑ ↑ Golgi apparatus, ↑ granular endoplasmic reticulum. Glutaraldehyde–osmium fixation: stained with uranium and lead. × 6900. (Photo: J. P. Thiery.)

Besides lung metastases, additional metastases were found in paravertebral lymph nodes (20%), liver (10%), kidney (10%) and adrenal glands (10%).

*Kinetics*

The mean doubling time of all bone tumours was 12·78 days (Table I). The difference between osteosarcoma and angiosarcoma, and between rats with or without metastases is not statistically significant.

Cell number doubling time of lung metastatic cells *in vitro* are presented in Table II. The doubling time (h) was different from one cell line to another, and there was no correlation between the doubling time of tumours *in vivo* and the doubling time of derived lung metastatic cells *in vitro*.

*Radioactivity*

Data on initial and final radioactivity are shown in Table III. The diffusion of radioactivity in liver and kidney was extremely limited.

FIG. 4.—Histology of undifferentiated angiosarcoma (lung metastasis). H.E. ×400.

TABLE II.—*Growth characteristics of $^{144}Ce$-induced primary tumours and derived lung metastatic cell cultures*

| Code | Histology of primary tumour | Doubling time (days) | Code of culture | Doubling time (h) |
|---|---|---|---|---|
| 30448 | Osteogenic osteosarcoma | 12·9 | HOM 01 | 20·5 |
| 30478 | Osteochondro-sarcoma | 11·4 | HOM 02 | 14·7 |
| 30472 | Undifferentiated osteosarcoma | 8·0 | HOM 03 | 16·8 |
| 30352 | Angiosarcoma | 7·3 | JAS 01 | 19·8 |
| 30335 | Angiosarcoma | 11·1 | JAS 02 | 16·8 |

The different doses of initial radioactivity in the range of 3·0 to 5·2 μCi did not influence the latent period between the injection of $^{144}$Ce and the appearance of tumours, nor the histology of tumours nor the presence or otherwise of lung metastases.

### DISCUSSION

It has been shown by several authors that inhalation of $^{144}CeCl_3$ can induce bone tumours in rats and other animals (Moskalev *et al.*, 1969). However, the doses we have injected are much lower than in those studies and they give a more efficient induction of bone tumours. Since there is an extremely limited diffusion of

TABLE III.—*Mean radioactivity after inoculation of $^{144}Ce$ and on day of killing*

| | Initial radioactivity in the right hind leg (μCi) | Final radioactivity in the right hind leg (μCi) | Final radioactivity in the liver (μCi) | Final radioactivity in the kidney (μCi) |
|---|---|---|---|---|
| All bone tumours | 4·66 ± 0·79 | 1·41 ± 0·46 | 0·013 ± 0·009 | 0·005 ± 0·002 |
| Bone tumours with lung metastasis | 4·44 ± 1·25 | 1·54 ± 0·49 | 0·014 ± 0·008 | 0·005 ± 0·002 |
| Bone tumours without lung metastasis | 4·41 ± 0·79 | 1·53 ± 0·55 | 0·015 ± 0·010 | 0·004 ± 0·002 |
| Osteosarcoma | 4·75 ± 0·80 | 1·39 ± 0·50 | 0·013 ± 0·008 | 0·005 ± 0·002 |
| Angiosarcoma | 4·47 ± 0·76 | 1·32 ± 0·29 | 0·013 ± 0·009 | 0·005 ± 0·003 |

FIG. 5.—Histology of primary osteosarcoma. H.E. ×640.

FIG. 6.—Histology of osteosarcoma (lung metastasis). H.E. ×380.

radioactivity if $^{144}Ce$ is injected as hydroxide, tumours are produced only at the site of injection.

In previously reported experiments the i.m. injection of the isotope produced mainly soft-tissue tumours and only few bone tumours (Klein *et al.*, 1977). It is probable that this difference was due to radioactivity diffusion. In this experiment, the mean dose of radioactivity injected was lower than that used in the former experiment. There is, in any case, no indication that the doses of radioactivity within the range we used may play a role in determining tumour incidence or the histological type of tumours.

Kinetic data derived from our bone tumours are quite heterogeneous. However, this correlates well with the histological polymorphism of these tumours. The mean doubling time of bone tumours was shorter than in the previous experiments (12·78 days *vs* 17·4 days) but superior to the values for soft-tissue sarcomas (8 days). The fact that cell cultures of lung metastases showed different kinetic characteristics, and did not correlate with the kinetic behaviour of their primary tumours *in vivo*, can be attributed to the predominance of other malignant cell clones in metastases or in cell culture. It is a further argument for the hypothesis that factors other than histology influence kinetics, *e.g.* anatomical site, microenvironment, cell loss, presence of non-cycling cells *etc.*

As in their human counterparts, the histological classification of $^{144}Ce$-induced bone tumours was difficult. Several of these tumours have been maintained by serial transplantation up to the 5th generation. Transplants of osteosarcoma and to a lesser extent tumours which have been classified as angiosarcomas too,

FIG. 7.—X-ray photograph of a rat with osteogenic sarcoma. AP view of tumour on right tibia.

incorporate 85 strontium (Thiery *et al.*, in preparation).

These results indicate that this model of radio-induced malignant bone tumour is reproducible and can be used for physiopathological or clinical studies of osteosarcomas. Its characteristics (histology, lung metastases and growth kinetics) make it a good experimental model for human osteosarcoma, and the fact that the technical modification here described is capable of increasing by up to 90% the incidence of malignant bone tumours points out the value of this model for the study of problems related to human osteosarcoma, such as: adjuvant treatment of lung metastases, screening for drugs active on osteosarcomas, markers *etc.* Since cell cultures are easily obtained, isologous or homologous hosts may be transplanted both *in vitro* and *in vivo.* Details of the morphological, cytochemical, biochemical characteristics and the transplantability of these cell lines and their kinetics *in vivo* will be published (Thiery *et al.*, in preparation).

In conclusion, we feel that we now possess a good model for studying *in vitro* and *in vivo* the multiple physiopathological and therapeutical problems of human osteosarcomas.

This investigation was supported by D.G.R.S.T. grant 76.7.1671. H. Delbrück is a research fellow of the M. Scheel Cancer research foundation.

### REFERENCES

COBB, I. M. (1970) Radiation-induced osteosarcoma in the rat as a model for osteosarcoma in man. *Br. J. Cancer*, **24**, 294.

FINKEL, M. P. & BISKIS, B. O. (1968) Experimental induction of osteosarcomas. *Progr. Exp. Tumour. Res.*, **10**, 72.

GEDDES-DWYER, V., BOSANQUET, J. S., O'GRADY, R. L. & CAMEROUN, D. A. (1974) Transplantation and tissue culture studies of radiation induced osteosarcoma in rat. *Pathology*, **6**, 71.

KLEIN, B., PALS, S., MASSE, R. & 5 others (1977) Studies of bone and soft-tissue tumours induced in rats with radioactive cerium chloride. *Int. J. Cancer*, **20**, 112.

LOUTIT, J. (1976) Vasoformative non-osteogenic (angio) sarcomes of bone-marrow stroma due to strontium-90. *Int. J. Radiat.*, **30**, 359.

MAZABRAUD, A. (1975) Production expérimentale de sarcomas osseux chez le lapin per injection unique locale de Béryllium. *Bull. du Cancer*, **62** (1), 49.

MOSKALEV, Y. I., STRELTSOVA, V. N. & BULDAKOV, L. A. (1969) Late effects of radionuclide in delayed effects of bone seeking radionuclides. *Symp. Proc., Sunvalley, Idaho University of Utah*, 439.

SOLHEIM, W. (1977) Development of osteosarcoma in rats after irradiation. *Acta Radiol.* [*Ther.*] (Stockh.), **5**, 433.